# Application of MRI Radiomics-Based Machine Learning Model to Improve Contralateral BI-RADS 4 Lesion Assessment

**DOI:** 10.3389/fonc.2020.531476

**Published:** 2020-10-29

**Authors:** Wen Hao, Jing Gong, Shengping Wang, Hui Zhu, Bin Zhao, Weijun Peng

**Affiliations:** ^1^ Department of Radiology, Fudan University Shanghai Cancer Center, Department of Oncology, Shanghai Medical College, Fudan University, Shanghai, China; ^2^ Shandong Medical Imaging Research Institute, Shandong University, Jinan, China

**Keywords:** MRI, contralateral breast cancer, radiomics, machine learning, Breast Imaging Reporting and Data System category 4

## Abstract

**Objective:**

This study aimed to explore the potential of magnetic resonance imaging (MRI) radiomics-based machine learning to improve assessment and diagnosis of contralateral Breast Imaging Reporting and Data System (BI-RADS) category 4 lesions in women with primary breast cancer.

**Materials and Methods:**

A total of 178 contralateral BI-RADS 4 lesions (97 malignant and 81 benign) collected from 178 breast cancer patients were involved in our retrospective dataset. T1 + C and T2 weighted images were used for radiomics analysis. These lesions were randomly assigned to the training (n = 124) dataset and an independent testing dataset (n = 54). A three-dimensional semi-automatic segmentation method was performed to segment lesions depicted on T2 and T1 + C images, 1,046 radiomic features were extracted from each segmented region, and a least absolute shrinkage and operator feature selection method reduced feature dimensionality. Three support vector machine (SVM) classifiers were trained to build classification models based on the T2, T1 + C, and fusion image features, respectively. The diagnostic performance of each model was evaluated and tested using the independent testing dataset. The area under the receiver operating characteristic curve (AUC) was used as a performance metric.

**Results:**

The T1+C image feature-based model and T2 image feature-based model yielded AUCs of 0.71 ± 0.07 and 0.69 ± 0.07 respectively, and the difference between them was not significant (P > 0.05). After fusing T1 + C and T2 imaging features, the proposed model’s AUC significantly improved to 0.77 ± 0.06 (P < 0.001). The fusion model yielded an accuracy of 74.1%, which was higher than that of the T1 + C (66.7%) and T2 (59.3%) image feature-based models.

**Conclusion:**

The MRI radiomics-based machine learning model is a feasible method to assess contralateral BI-RADS 4 lesions. T2 and T1 + C image features provide complementary information in discriminating benign and malignant contralateral BI-RADS 4 lesions.

## Introduction

Breast magnetic resonance imagery (MRI) demonstrates a high sensitivity for contralateral occult malignancies on mammography or ultrasonography. It is widely used for pre-treatment evaluation, especially for patients preparing for breast-conserving surgery. This may be the reason for the higher incidence of contralateral detection in recent decades. Primary breast cancer patients have intermediate risk for contralateral malignancies (1, 2). The risk is 2–6 times that of the risk for a woman first developing a breast cancer (3). Therefore, the likelihood of malignancy for a suspicious contralateral lesion may be different from that of an ipsilateral lesion. Moreover, the knowledge of an extra finding changes the treatment plan and causes more patient anxiety. A precise and personalized diagnostic strategy should be established for this unusual situation.

According to the American College of Radiology (ACR) guidelines, Breast Imaging Reporting and Data System (BI-RADS) category 5 refers to a malignancy likelihood of 95% or greater (4) and the positive predictive value of this category is as high as 97.5% (5); therefore, it is not a major source of misdiagnosis. However, a lesion classified as BI-RADS category 4 corresponds to a wide likelihood of malignancy, ranging from 2% to 95% (4). Breast MRI is known to be highly sensitive, but there is significant overlap between the imaging characteristics of some atypical malignant lesions and other benign lesions (6). These lesions, whether benign or malignant, could easily be categorized as BI-RADS 4 and recommended for invasive biopsy. As the range of positive predictive values for MRI-guided biopsies (19.5 to 42.7%) shows (6–9), many patients received unnecessary invasive procedures. By improving assessment for BI-RADS 4 lesions, benign lesion may be correctly recognized, and unnecessary biopsy avoided.

Unlike the traditional practice of using medical images solely for visual interpretation, radiomics transmits digital medical images into mineable data by extracting abundant quantitative features from regions of interest. These features contain comprehensive tumor characterization information, such as tumor size, shape, intensity, and texture. Radiomics data can be applied to build descriptive or predictive models that correlate quantitative image features with phenotypes or gene-protein markers, potentially assisting in cancer detection/diagnosis, treatment response prediction, and prognosis assessment. Previous studies have shown that a radiomics method could aid in the diagnosis, molecular subtyping, prognosis, and treatment response prediction for breast cancer patients (10–13).

To improve the assessment of BI-RADS 4 lesions, some researchers developed prediction models using specific imaging features or multi-parameter MRI data (14–16). However, these studies only investigated the traditional imaging features, which were defined by radiologists subjectively. Whether or how the radiomics method can be used to predict malignancy for contralateral BI-RADS 4 lesions has not been explored. The purpose of this study was to investigate and explore the possibility of using an MRI radiomics-based machine learning model to improve the assessment and diagnosis for contralateral BI-RADS 4 lesions in primary breast cancer patients.

## Material and Methods

### Patient Selection

Institutional review board approval was obtained for this study and the need for informed patient consent was waived due to the study’s retrospective nature.

A total of 24,588 consecutive pre-treatment breast dynamic MRI examinations performed between January 2016 and December 2018 were retrospectively reviewed by our imaging data system The inclusion criteria were as follows: (a) primary breast cancer was detected by self-examination, clinical palpation, or imaging examination; (b) pre-treatment breast MRI revealed a contralateral BI-RADS 4 lesion, for which the histopathological subtype was confirmed by surgery or biopsy; (c) no history of breast cancer.

### MRI Acquisition

All breast MRI examinations were performed using a 3.0T (Skyra, Siemens, Munich, Germany) scanner using a dedicated breast coil with the patient in a prone position. For each case, there was a fat-saturated T2-weighted sequence (TR 3,570 ms, TE 69 ms, slice thickness 5 mm, FOV 360 mm, matrix 384*384), and fat-saturated T1-weighted dynamic sequences (TR 4.5 ms, TE 1.6 ms, slice thickness 2.2 mm, FOV 360 mm, matrix 384*384), including one pre-contrast and five dynamic post-contrast series obtained following intravenous administration of gadopentetate dimeglumine (Magnevist, Bayer Health Care, Berlin, Germany), which was power injected (Spectris Solaris EP, Medrad, Pittsburgh, PA, USA) at a dose of 0.1 mmol/Kg at a rate of 2 mL/s. A total volume of 20 mL saline was used to flush the contrast medium.

### Pathology

Pathology diagnosis was retrieved from the electronic records at our institute. The available reports were divided into malignant and benign categories. Lesions considered to be high risk in nature (atypical findings, lobular neoplasia, complex sclerosis, or papillary lesions) were categorized as benign. In cases with mixed histological features, the most aggressive pattern was used as the grouping indicator.

### Patients’ Grouping

To train and test the classification model, 178 patients were randomly assigned to a training dataset (n = 124, 70%) and an independent testing dataset (n = 54, 30%). The basic information of patients, including age, menopause status, family history of breast cancer and breast density was compared between the training and testing datasets. A chi-square test and an independent sample t test were used for appropriate data type. All above statistical analyses were performed with IBM SPSS 21.

### Diagnostic Scheme Build-Up

**Figure 1:**
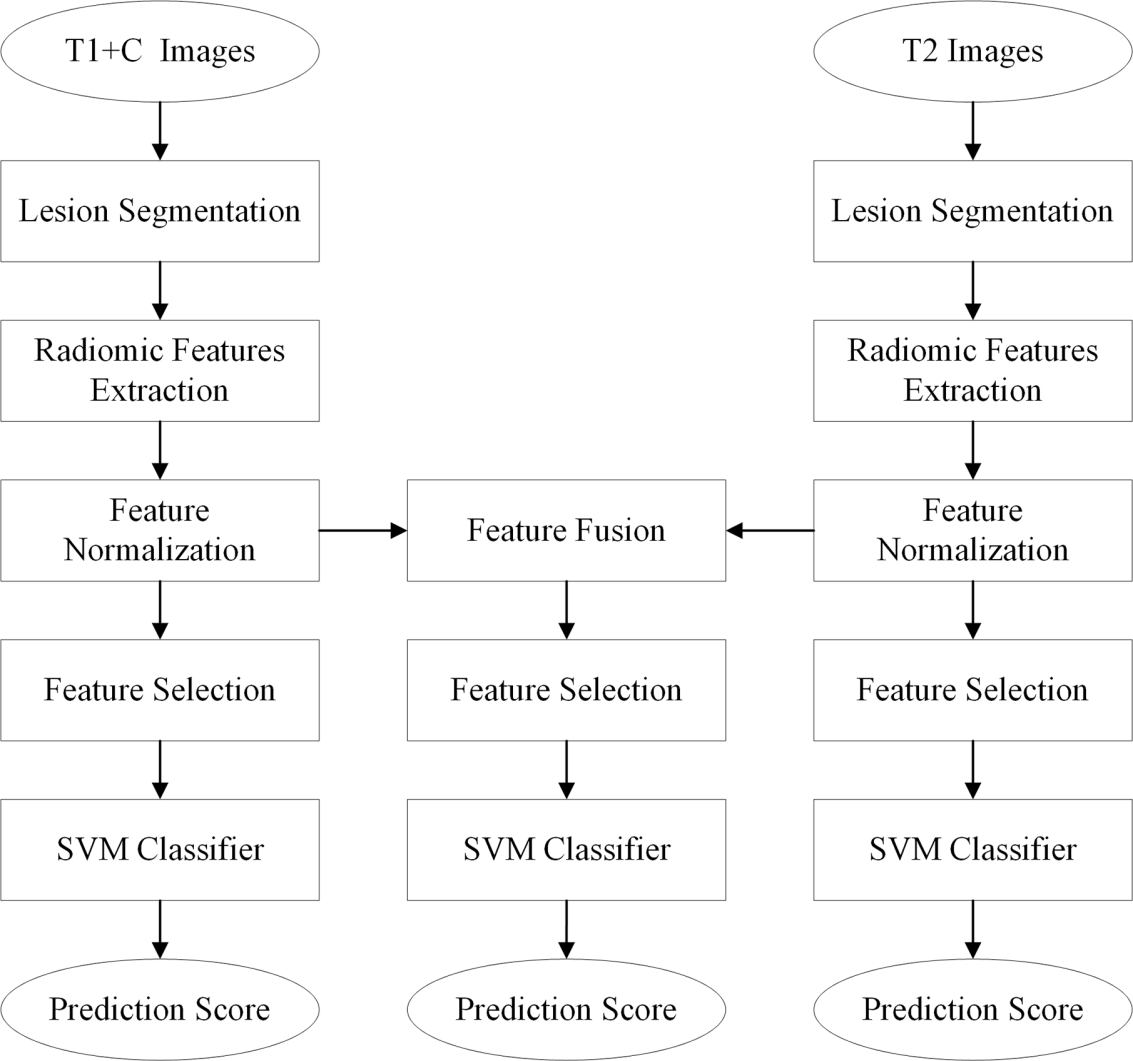
Flowchart of the proposed radiomics analysis method.

The diagnostic schemes based on the T1+C and T2 images were developed to respectively predict and assess the malignancy likelihood of suspicious contralateral lesions. Since T1 and T2 images represent different tumor phenotypes, an imaging feature fusion method was used to combine the T1+C and T2 radiomic features (**Figure 1**).

**Figure 2:**
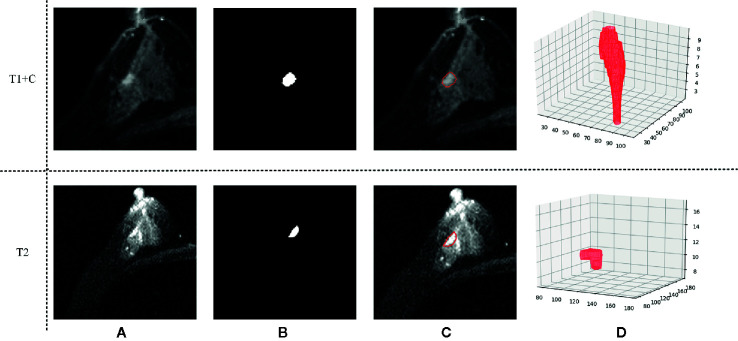
An example of the segmentation result. **(A)** Shows the original T1 + C/T2 image, **(B)** shows the masks generated by our semi-automatic segmentation method, **(C)** shows the final segmentation result, and **(D)** shows the 3D tumor volume.

Three-dimensional (3D) semi-automatic segmentation was performed on the T1+C and T2 images (17). All center positions of lesions were first delineated by a radiologist on T1+C and T2 scans. Using the marked lesion center point as the initial seed point, a 6-connected neighborhood 3D region growing method was used to roughly segment the lesion boundary. In the region growing algorithm, a threshold value of 90 was used to compare voxel value with seed point. Then, a level set algorithm used geodesic active contouring to refine the lesion boundary. In this process, a gradient magnitude recursive Gaussian image filter configured with δ of 0.5 was first used to filter the initial ROI image. The propagation scaling value of 1.0, curvature scaling value of 0.5, advection scaling value of 1.0, maximum RMS error value of 0.005, and iteration number of 1,000 were configured to build the geodesic active contour level set image filter. Finally, a 3D morphological closing operator and a flood-fill algorithm were applied to fill the small holes in the lesion masks (18). **Figure 2** shows an example of the segmentation result.

Due to the ununified spacing of T1 + C and T2 images collected from different MRI scanners, a cubic B-spline interpolation was applied to resample the images. After image resampling, all the T1 + C and T2 images were standardized to a spacing of (1 mm, 1 mm, 1 mm). To decode the breast tumor imaging phenotypes, a radiomic feature analysis method was applied to characterize the lesion’s imaging features. A total of 1,046 radiomic features were extracted from segmented lesions. Among these features, 258 LoG features were computed using the Laplacian of Gaussian filter with sigma values of 1, 2, and 3; 688 wavelet features were obtained by filtering the original image with a wavelet filter; and 14 shape features, 18 histogram features, and 68 texture features were involved. These texture features consisted of 22 gray-level co-occurrence matrix texture features, 14 gray-level dependence matrix texture features, 16 gray-level run length matrix texture features, 16 gray-level size zone matrix texture features, and 5 neighboring gray-tone difference matrix texture features.

**Figure 3:**
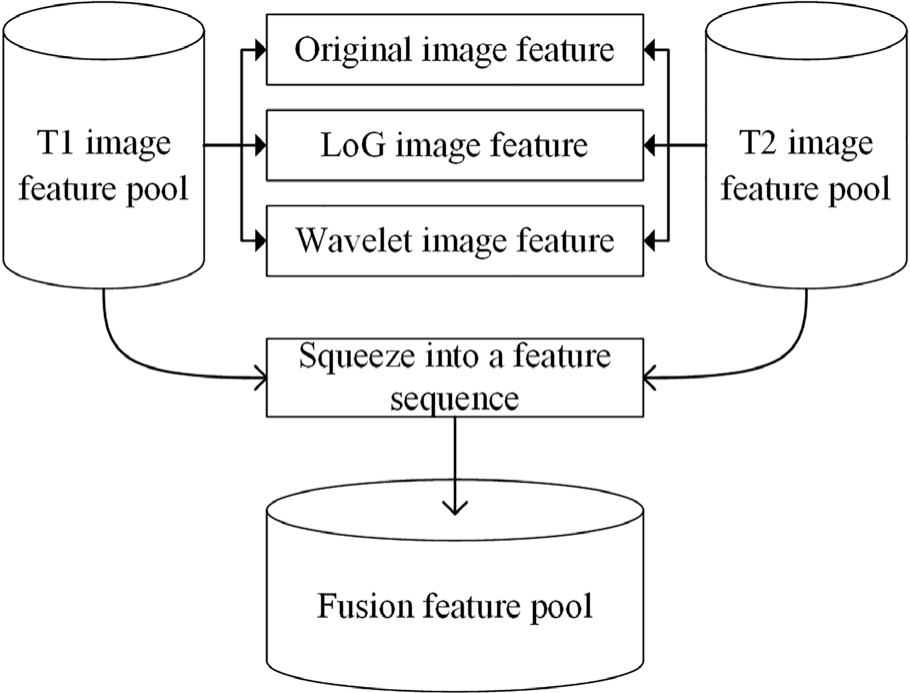
The workflow of the image feature fusion process.

Before scheme building, each radiomic feature was normalized by scaling to [0, 1]. A relief feature selection method was used to remove the low-performance features and reduce the dimensionality of feature space. To avoid the overfitting problem in the classifier training/testing process, 10% of the sample size was empirically selected as the maximum value of the selected feature number. Then, a least absolute shrinkage and selection operator (Lasso) feature selection method was used to choose the optimal imaging features by evaluating the classification accuracies of our scheme. The penalty term value of the Lasso feature selector α was set as 0.001. With Lasso, the higher the alpha parameter, the fewer features selected. For a good choice of alpha, the Lasso can fully recover the exact set of non-zero variables using only few observations, provided certain specific conditions are met. To obtain an optimal alpha, we used a series of values range from 0.0001 to 1.0 with a step of 0.1 to build feature selectors. By evaluating the model performance with different feature selectors, we selected alpha = 0.001 with the highest model performance as the optimal one. To build a classification model, a support vector machine (SVM) classifier configured with a radial basis function (RBF) kernel was trained and tested using the selected features. To build a fusion model, the T1 + C and T2 image features were merged to build a whole imaging feature pool. In this process, the initial T1 + C and T2 image features (involving original image feature, LoG image feature, and wavelet image feature) were squeezed into a feature sequence to build a fusion feature pool. **Figure 3** shows the workflow of the image feature fusion process. Next, the same feature selection method and machine-learning classifier were applied to build a classification model.

### Performance Evaluation

The AUC values of T1 + C, T2, and fusion schemes were computed by applying a maximum likelihood-based receiver operating characteristic (ROC) fitting program (ROCKIT, http://metz-roc.uchicago.edu/MetzROC/software/, University of Chicago). The comparison of AUC values was performed between T1 + C, T2, and the fusion scheme, and p-value was corrected with the Bonferroni method. All above computation processes and data analyses were processed in Python 3.6 using a computer with Intel Core i7-8700 CPU 3.2GHz × 2, 16 GB RAM. Several open source libraries, including pyradiomics, SimpleITK, scikit-image, matplotlib, and scikit-learn, were applied in this study. In the model development and validation process, the functions in python libraries were configured with the default parameters. Thus, our proposed model was straightforward and could be easily applied and/or validated in future studies.

## Results

### Patients’ Basic Information

A total of 178 women were recruited for this study. The mean age was 51 years (range, 25–78 years). **Table 1** provides demographic details for the patient cohort.

**Table 1:** Basic information for the patient cohort.

Characteristic	Training dataset (N = 124)	Testing dataset (N = 54)	Total	P value^a^
Age (y)				
Mean ± SD	49.6 ± 11.44	53.2 ± 11.48	50.7 ± 11.54	0.057^b^
Range	25–78	28–78	25–78	
Menopausal status				
Premenopausal	89	32	121	0.117
Postmenopausal	35	22	57	
Family history of breast cancer				
Yes	21	8	29	0.827
No	103	46	149	
MRI breast density				
1	3	2	5	0.150
2	21	16	37	
3	86	28	114	
4	14	8	22	

a P values were calculated by chi-square test.

b P value was calculated by independent sample t test.

Patients underwent breast MRI examination for pretreatment evaluation (n = 92), problem solving for an equivocal mammogram or ultrasound ﬁnding (n = 73), high-risk screening (n = 5), clinical symptoms with negative conventional imaging (n = 5), and axillary metastasis looking for a primary breast cancer (n=3).

Of 97 contralateral malignant lesions, simple mastectomy was performed on 59 lesions, breast conserving surgery on 16 lesions, and modified radical mastectomy on eight lesions. The remaining nine lesions were confirmed by mammography, ultrasound, or MRI-guided core biopsy because these patients were undergoing neoadjuvant chemotherapy (NAC). A total of 19 patients received secondary surgery due to underestimation of biopsy or pathological results during operation.

Of 81 contralateral benign lesions, quadrant resection was performed on 69 lesions, while simple mastectomy was performed on five lesions. The remaining seven lesions were confirmed by biopsy.

### Pathological Findings

The pathological distribution of primary lesions was invasive ductal carcinoma (IDC) in 130 patients, ductal carcinoma *in situ* (DCIS) in 35 patients, introductal papillary carcinoma in four patients, mucinous carcinoma in three patients, invasive micropapillary carcinoma in two patients, encapsulated papillary carcinoma in two patients, neuroendocrine carcinoma in one patient, and invasive apocrine carcinoma in one patient. The average size of primary cancers was 3.4 cm (ranging from 0.3 cm to 9.5 cm). Among the 178 contralateral lesions, 97 were shown to be malignant, including 40 IDCs, 34 DCISs, nine invasive lobular carcinomas, six introductal papillary carcinomas, two mucinous carcinoma, two lobular carcinomas in situ, two encapsulated papillary carcinomas, one neuroendocrine carcinoma, and one invasive apocrine carcinoma, for a malignancy rate of 54.5%. The average size was 3.7 cm (ranging from 0.6–10 cm). The remaining 81 were classified as benign, including 45 pure adenoses, 19 intraductal papillomas, 10 sclerosing adenoses, five fibroadenomas, one lobular neoplasia, and one phyllodes tumor. The average size was 2.05 cm (range, 0.7–7.8 cm).

### Radiomics Analysis and Diagnostic Performance

**Figure 4:**
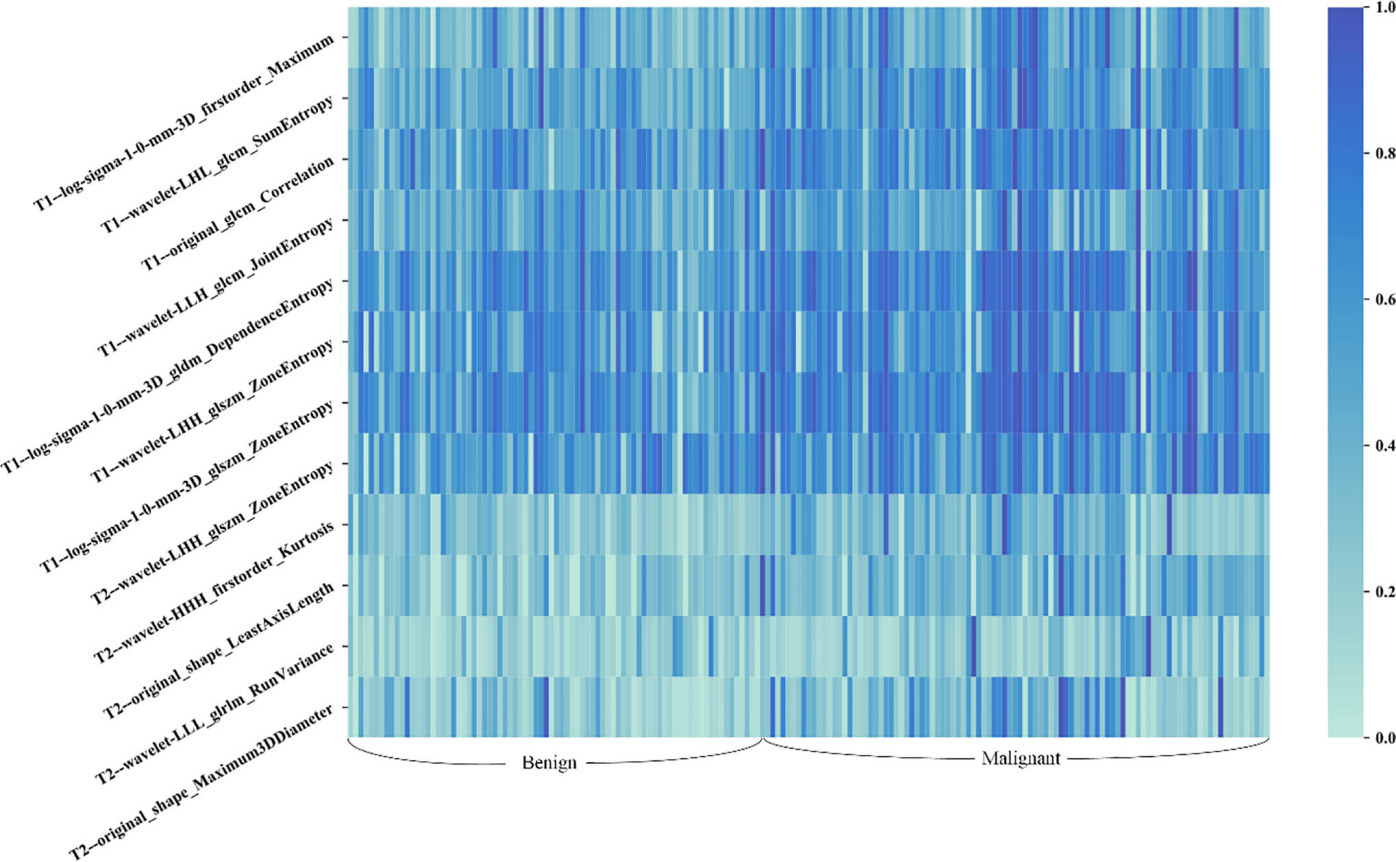
Heat map of the selected radiomic features for T1 + C and T2 schemes. Each row of the heat map represents a radiomic feature and each column represents a patient. Different shades of blue represent different values of radiomic features. The difference in T1 + C feature values between benign and malignant lesions was slightly more distinct than that of T2 features.

A total of seven radiomics features, including three wavelet features, one texture feature, and three LoG features, were selected from the initial T1 + C imaging feature pool. Five features, including three wavelet features, and two shape features, were frequently selected from the initial T2 imaging feature pool. **Figure 4** shows the heat map of the 12 selected imaging features.

**Table 2** compares the performances of the three machine learning models. The accuracy and sensitivity scores under two specificity values, 71.4% and 78.6%, were listed and compared. The fusion image feature model yielded an accuracy of 74.1%, which was higher than that of the T1 + C (66.7%) and T2 (59.3%) image feature models. Meanwhile, the fusion model obtained sensitivity scores of 76.9% and 65.4% under the specificity values of 71.4% and 78.6%, respectively, which were higher than the sensitivity scores of the T1 + C model (65.4% and 30.8%) and T2 model (69.2% and 57.7%).

**Table 2:** Comparisons of classification accuracy and sensitivity scores under two specificity values generated by three classification models.

Classification model	Accuracy (%)	Sensitivity (%)(Specificity=71.4%)	Sensitivity (%)(Specificity=78.6%)
T1 + C features	66.7	65.4	30.8
T2 features	59.3	69.2	57.7
Fusion features	74.1	76.9	65.4

T1 + C: T1 weighted image with contrast medium.

**Figure 5:**
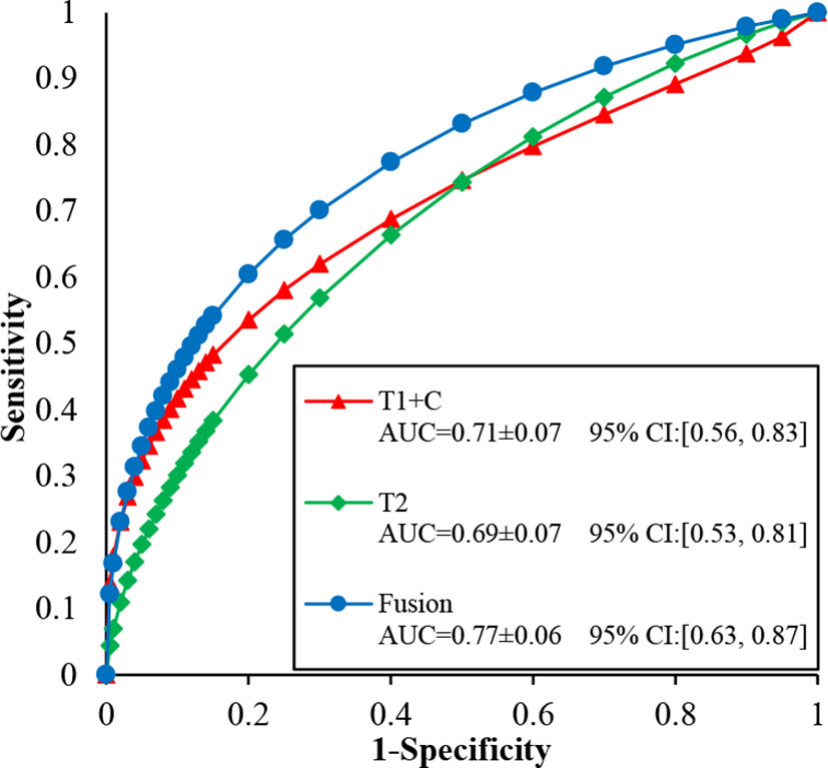
Comparison of ROC, AUC, and 95% confidence interval (CI) values generated using T1 + C, T2, and fusion diagnostic scheme, respectively.

**Figure 5** illustrates the ROC, AUC, and 95% confidence interval (CI) values of the T1 + C, T2, and fusion schemes, respectively. Compared with the T2 scheme, the T1 + C scheme yielded a slightly higher AUC value when tested on the same dataset (0.71 ± 0.07 vs. 0.69 ± 0.07, P > 0.05). The fusion scheme generated the best AUC value, 0.77 ± 0.06, which was significantly higher than the AUCs of the T1 + C and T2 schemes (P < 0.001, <0.05/3).

## Discussion

It is important to determine the contralateral situation for a patient with primary breast cancer. For simultaneous bilateral breast cancer (SBBC) patients, the actuarial survival rates at five years were lower, and the distant metastasis and unfavorable disease-specific survival were higher than those of patients with unilateral cancer (18, 19). In essence, contralateral cancer detection is a form of high-risk screening. At present, breast MRI has become the main tool for pre-treatment contralateral evaluation for recently diagnosed breast cancer patients (20). Breast MRI depicts occult contralateral disease in 5.5–9.3% of women with known unilateral breast cancer; 37–48% of these findings (2–4%) are malignant (20, 21).

Because there are two lesions present in one patient, the clinical considerations for SBBC are more complicated than those for unilateral breast cancer. However, detection of suspicious contralateral lesions is more complicated than detecting their unilateral counterparts. Previous studies reported that, compared with primary tumors, contralateral malignant tumors consist of more DCISs and uncommon pathological subtypes (22, 23). In this study, we observed a large proportion of DCIS and many uncommon malignant lesions, such as encapsulated papillary carcinoma, neuroendocrine carcinoma, and invasive apocrine carcinoma. These malignant conditions usually demonstrate atypical MRI features, which partially overlap with those of some benign lesions (24–27). However, over 55% (45/81) of benign lesions in this study were proven to be adenoses, which are benign lesions sometimes demonstrating suspicious features on breast MRI, but requiring no specific treatment because they pose a small risk for future cancer development (28). These unusual conditions, benign or malignant, are easily assigned into the BI-RADS 4 category and recommended for biopsy in accordance with ACR BI-RADS guidelines. However, for a patient who has a highly suspicious lesion in one breast, biopsy for a less-suspicious lesion in the contralateral breast may be considered time-consuming and expensive. In this study, 91% (162/178) of our collected patients skipped biopsy and chose resection directly, and 19 patients received secondary surgery due to biopsy underestimation or pathological results during the operation. To help patients and clinicians choose the most precise treatment plan for an initially detected suspicious contralateral lesion, a more accurate assessment method is needed.

Radiomics has proven to be a promising tool for many clinical purposes. In this study, we first used radiomics to improve the assessment of contralateral BI-RADS 4 lesions. A total of 1,064 radiomics features were initially extracted from T2 and T1 + C images. After removing redundant features, only seven features were ultimately used to build the T1 + C scheme, and five were used to build the T2 scheme. The selected features of the two schemes were different, and indicated that T1 + C and T2 images may represent different phenotypes of breast lesions. T2 images reflect not only the presence of the tumor tissue, but also peri-tumor edema (29). A previous study proved that features extracted from T2 images were associated with the Ki-67 status (30) and the pathological response to neoadjuvant chemotherapy in breast cancer (31). The signal hyperintensity of T1 + C images contains anatomic and vascular information that is crucial for discriminating benign and malignant lesions. As that the resolution and slice thickness of T1 + C images are generally superior to routine T2 images, features extracted from T1 + C images were commonly used in most previous studies (10, 12, 13). In the current study, T2 and T1 + C features were used to build diagnostic schemes, and ROC analysis revealed that the two schemes generated similar AUC values. After fusing these two types of imaging features, the prediction performance significantly improved. These results indicated that T2 and T1 + C features provide complementary information useful in discriminating benign and malignant contralateral BI-RADS 4 lesions. In the further studies, both T2 and T1 + C images should be used for model building.

Radiomics classifiers predict the likelihood of malignancy for BI-RADS 4 lesions. Ideally, a competent classifier provides a low probability for a benign lesion, enabling suspension of invasive procedures in favor of a cautious follow-up, and provides a high probability for a malignant lesion, ensuring that it will be recommended for biopsy or surgery and avoiding the need for a second surgery. In the current study, the fusion scheme combining T1 + C and T2 features attained a strong AUC value of 0.77 and an accuracy of 74.1%. Although the fusion model still requires improvement before it can be used to support clinical decision-making, the model has demonstrated its promise. Moreover, this method is objective because it is not affected by the existence of a primary lesion.

This study had several limitations. First, the number of patients was relatively small for radiomics analysis. Whether these samples can sufficiently represent the diverse contralateral BI-RADS 4 lesion population is unknown. The reproducibility and robustness of the reported results need to be further validated with large datasets. This was the main limitation of this study. The incidence of bilateral breast cancers was relatively low. However, for the sake of data consistency, we restricted our collection to patients who were examined using the same scanner. Second, only T2 and one phase of T1 + C images were used for radiomic feature extraction. Considering more inconsistency may be introduced by varying acquisition parameters and times of DWI and dynamic sequences, ADC maps and multi-phase contrasted images were not included in this study. Since the combined radiomics features from DCE-MRI and ADC data may serve as potential predictor markers (32), the discriminating efficiency will hopefully be further improved by adding other types of images for radiomic feature extraction. Third, the boundaries of breast lesions may be imprecise when only using a 3D semi-automatic segmentation method. Thus, developing a more accurate and robust segmentation method is one of our goals for future studies.

In conclusion, the MRI radiomics-based machine learning model is a feasible tool for contralateral BI-RADS 4 lesion assessment. T2 and T1 + C features provide complementary information useful in discriminating benign and malignant contralateral BI-RADS 4 lesions.

## Data Availability Statement

The datasets generated for this study are available on request to the corresponding author.

## Ethics Statement 

The studies involving human participants were reviewed and approved by Institutional review board of Fudan university Shanghai cancer center. Written informed consent for participation was not required for this study in accordance with the national legislation and the institutional requirements.

## Author Contributions

WH and JG conceived and designed the study, collected, analyzed, and interpreted the data, prepared the draft. Authors WH and JG had the equal contribution to this study and they shared the first authorship of this manuscript. SW and HZ interpreted data analysis and carried out clinical revision of the data. BZ and WP reviewed and revised the manuscript. All authors contributed to the article and approved the submitted version.

## Funding

This study has received funding by the National Natural Science Foundation of China (61731008).

## Conflict of Interest

The authors declare that the research was conducted in the absence of any commercial or financial relationships that could be construed as a potential conflict of interest.
